# Claudin expression profile in flat wart and cutaneous squamous cell carcinoma in epidermodysplasia verruciformis

**DOI:** 10.1038/s41598-020-66065-y

**Published:** 2020-06-09

**Authors:** Lana Luiza da Cruz Silva, Walmar Roncalli Pereira de Oliveira, Naiura Vieira Pereira, Ilana Halpern, Claudia Kwei-Fong Dai Tanabe, Mayra Servilha Grion Mattos, Mirian N. Sotto

**Affiliations:** 1 Departament of Dermatology, Faculdade de Medicina da Universidade de São Paulo, São Paulo, SP Brazil; 2 Departament of Pathology, Faculdade de Medicina da Universidade de São Paulo, São Paulo, SP Brazil

**Keywords:** Squamous cell carcinoma, Oncogenesis

## Abstract

Epidermodysplasia verruciformis (EV) is a genodermatosis related to human beta-papillomavirus (beta-HPV), with a high risk of cutaneous squamous cell carcinoma (cSCC). Claudins are transmembrane proteins expressed in epithelia and may be altered during carcinogenesis. For a better understanding of the role of beta-HPV in cutaneous carcinogenesis, this claudin expression study was conducted on lesions of patients with and without EV. In this study, claudins-1, -2, -3, -4, -5, -7 and -11 expressions were analyzed by applying the immunohistochemistry technique, in samples of 108 normal skin, 39 flat warts and 174 cSCC. The cSCC samples were organized in tissue microarrays. We found that claudin-1 and claudin-3 focal expressions were associated with cSCC (p < 0.001), and claudin-2 focal or negative expression with flat wart (p < 0.001), in EV and NEV (non-EV) groups. For claudin-5, EV group showed a lower chance of focal and negative expression (p < 0.001), and its negative expression was associated with flat wart (p < 0.001) and lower mean age (p < 0.001). Claudins-4, -7 and -11 showed a diffuse expression in almost all studied samples. Our findings suggest that claudin-5 increased expression observed on normal skin, flat wart and cSCC showed association with EV. Claudin-1 and -3 down expression were also observed, but they could not be related to beta-HPV infection.

## Introduction

Epidermodysplasia verruciformis (EV) is a rare genodermatosis, with multifactorial etiopathogenesis, which results in abnormal susceptibility to a specific group of human papillomavirus genotypes (beta-HPV)^1,2^. EV patients develop skin lesions throughout their lives, characterized by pityriasis versicolor-like macules, seborrheic keratosis and flat warts^1,3,4^. Lesions may undergo malignant transformation in up to 50% of the cases, mainly in sun-exposed areas. The most frequent tumors are Bowen’s disease and cutaneous squamous cell carcinoma (cSCC)^1,2,4^. The identification of HPV-5 genome in skin cancer in EV patients was the first evidence of HPV involvement in human cancer and since then, EV has been considered a model of study of viral oncogenesis in humans^1,5,6^.

However, oncogenesis in EV is still not fully understood. Unlike genital carcinomas induced by alpha-HPV, beta-HPV DNA does not integrate into human genome^6^. Current data suggest that beta-HPV may act on an initial stage of skin carcinogenesis, by destabilizing the host genome, with synergistic cooperation between ultraviolet (UV) radiation and immunity impairment of the host^6^.

Changes in cell adhesion proteins, such as claudins, have been studied for a better understanding of viral oncogenesis. Formed by 27 isoforms, claudins are the major transmembrane proteins of the tight junctions (TJ), which are intercellular junctions located adjacent to the apical end of paracellular spaces^7^. Claudins are involved in the cytoskeletal maintenance, cell signaling and TJ permeability^8^. They are expressed in normal epithelium, where they show different profiles, which are responsible for the heterogeneity of paracellular characteristic among epithelia^9^. In epithelial tumorigenesis, both decreased and increased claudin expression have been associated with a biological behavior of the tumor, including involvement in survival and invasion processes of neoplastic cells^10,11^.

Nowadays, it remains unclear if there is a significant correlation between beta-HPV infection and claudins expression. Thus, we consider interesting to analyze claudins expression pattern in EV, throughout the progression of normal skin to flat wart and cutaneous SCC, comparing them to normal skin, flat warts and neoplastic processes from individuals without EV.

## Results

EV patients consisted of 14 males and 19 females and non-EV (NEV) were 63 males and 49 females. The NEV group had a mean age of 71.1 (ranging from 3–96 years old), older than the EV group (44.8, ranging from 8–70 years old) (p < 0.001).

**Figure 1:**
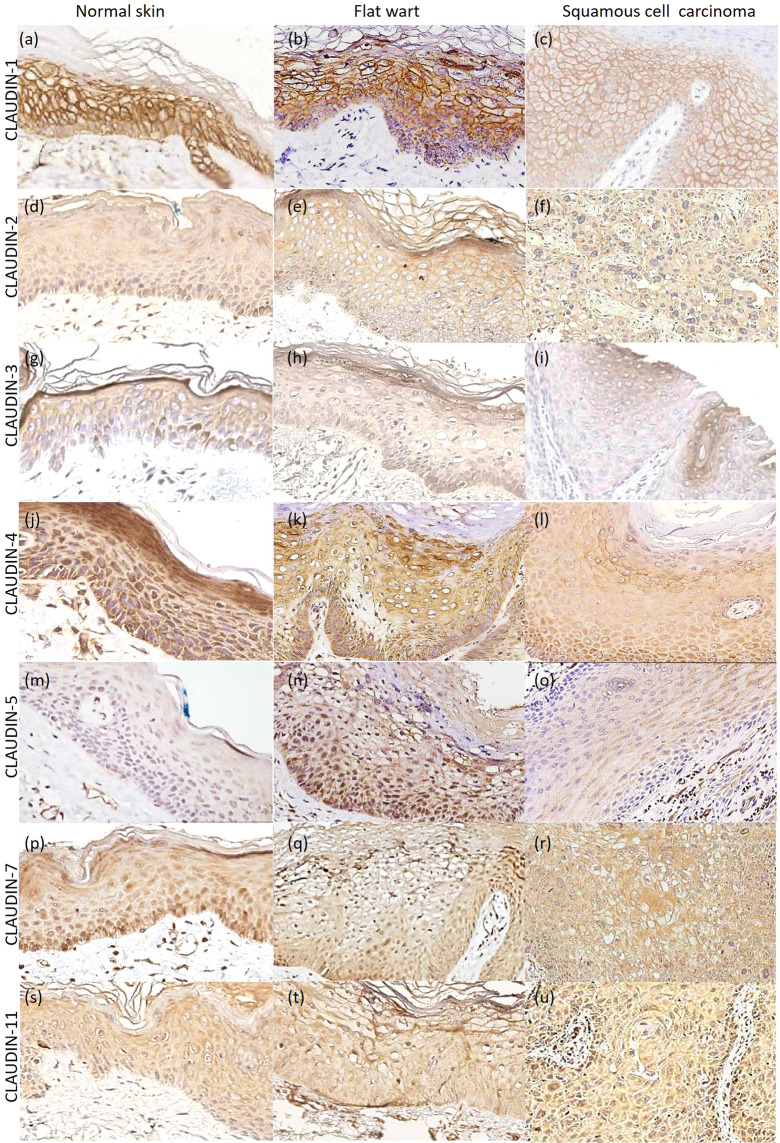
Epidermodysplasia verruciformis group: claudins expression in normal skin, flat wart and cutaneous squamous cell carcinoma (cSCC). (**a–c**) Claudin-1 membranous bound staining, with diffuse distribution in all histological types. (**d–f**) Claudin-2 diffuse expression, with membranous and cytoplasmatic immunostaining. (**g–i**) Claudin-3 diffuse expression in normal skin (upper layers) and flat wart, however with focal expression in cSCC. (**j–l**) Claudin-4 diffuse distribution, with cytoplasmatic immunostaining. (**m–o**) Claudin-5 focal expression in normal skin (upper layers), but with diffuse pattern in flat wart and cSCC. Diffuse expression of claudin-7 (**p–r**) and claudin-11 (**s–u**) in all histological types, with cytoplasmatic immunostaining. *Immunohistochemistry images photographed by the author in 2019*^57^.

**Figure 2:**
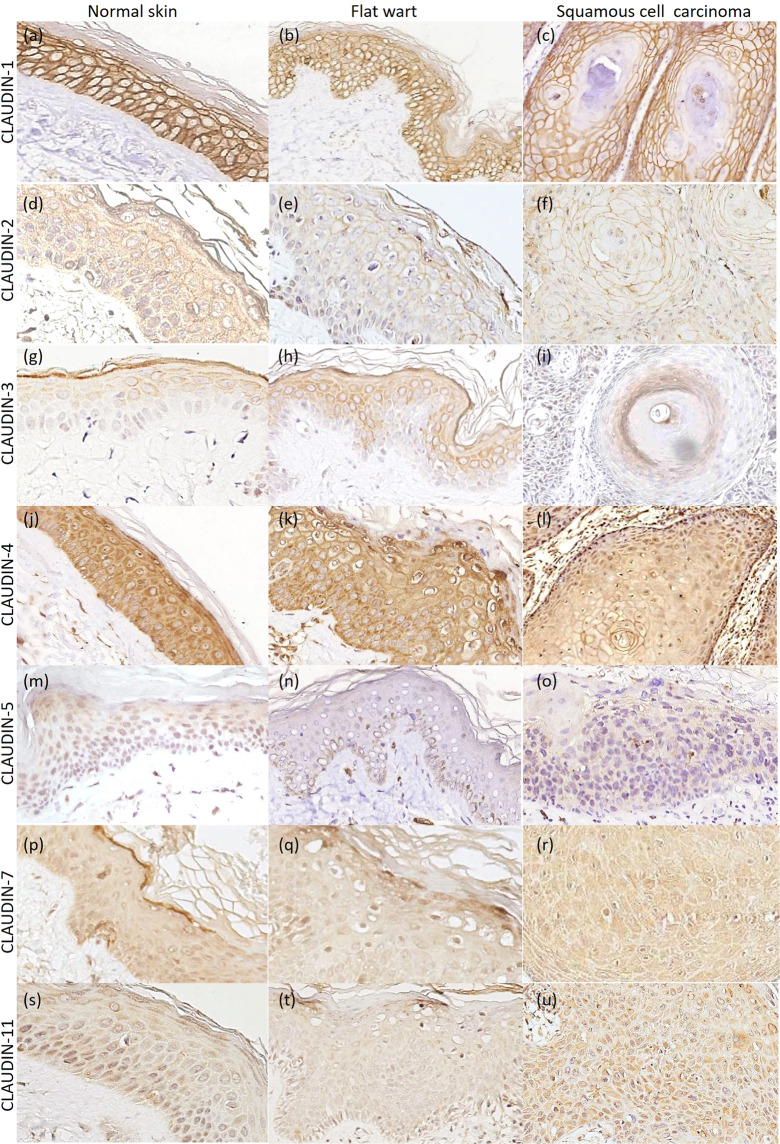
Not epidermodysplasia verruciformis group: claudins expression in normal skin, flat wart and cutaneous squamous cell carcinoma (cSCC). (**a–c**) Claudin-1 membranous bound staining, with diffuse distribution in all histological types. (**d–f**) Claudin-2 diffuse expression, with membranous and cytoplasmatic immunostaining. (**g–i**) Claudin-3 diffuse expression in normal skin (upper layers) and flat wart, however with focal expression in cSCC, with positivity at the center of the tumor islands. **(j–l)** Claudin-4 diffuse expression, with membranous and cytoplasmatic immunostaining. (**m–o**) Claudin-5 focal expression in normal skin (upper layers) and flat wart, but with diffuse distribution in cSCC. Diffuse expression of claudin-7 (**p–r**) and claudin-11 (**s–u**) in all histological types, with cytoplasmatic immunostaining. *Immunohistochemistry images photographed by the author in 2019*^57^.

**Figure 3:**
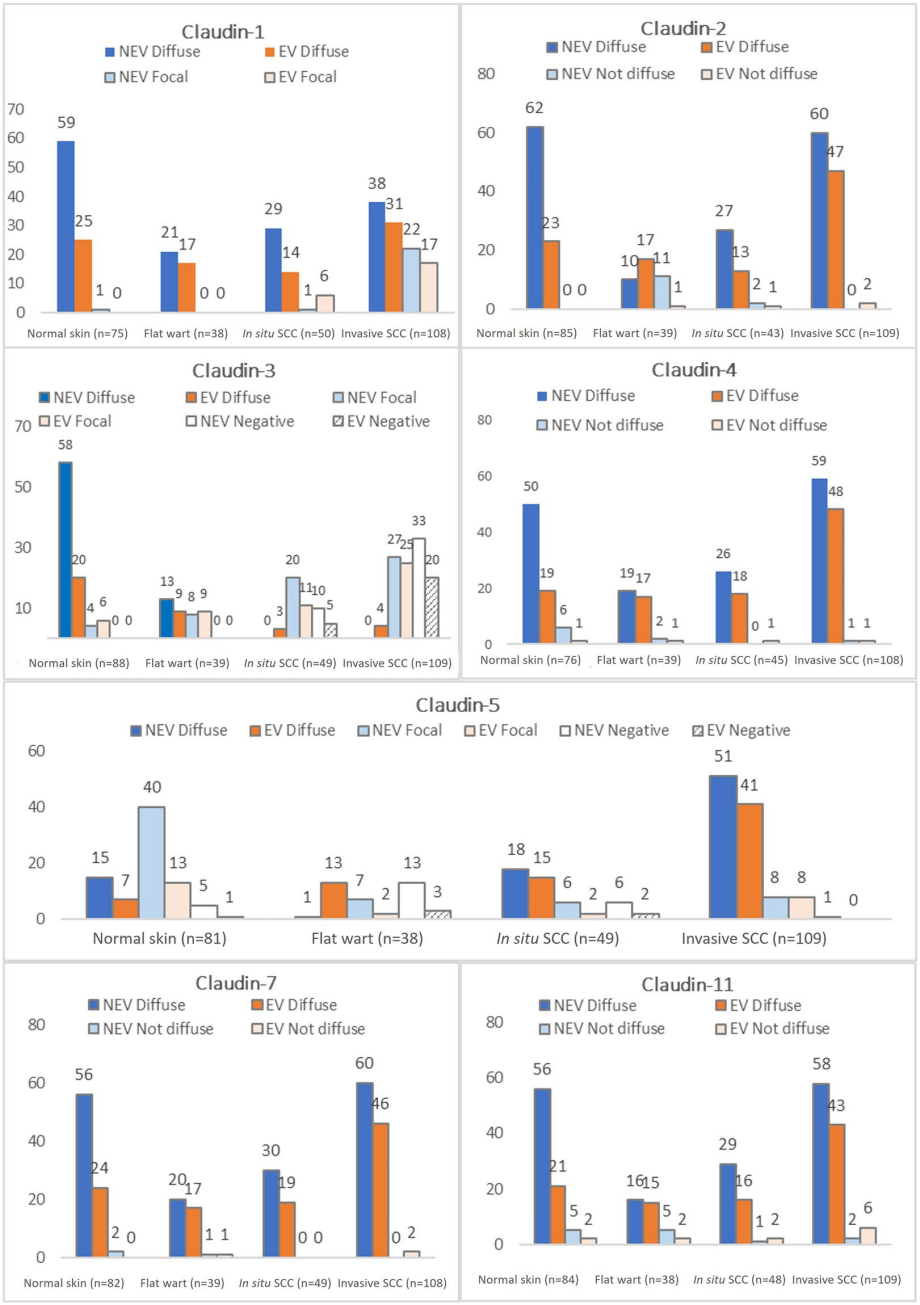
Expressions distribution of claudins-1, -2, -3, -4, -5, -7 and -11 in different histologic skin samples (normal skin, flat wart and cutaneous squamous cell carcinoma- cSCC), based on absolute frequency of the cases (n), comparing EV and NEV groups. According to these results, one may see claudins expression in cSCC’s progression. For example, invasive cutaneous squamous cell carcinoma expressed more cases of diffuse expression of claudin-5 than normal skin. EV: epidermodysplasia verruciformis; NEV: not epidermodysplasia verruciformis; cSCC: cutaneous squamous cell carcinoma.

Regarding the immunostaining site, claudin-1 exhibited membrane bound staining, although some cases with cytoplasmic expression were observed. The other claudins showed membranous and cytoplasmic staining. Claudin-3 also showed marked nuclear immunostaining, mainly in cSCC samples. Immunostaining of vascular endothelium was observed in claudin-5 and it was considered as tissue positive control of the reaction. Expression of claudins-1, -2, -3, -4, -5, -7 and -11 in normal skin, flat warts and cSCC, from EV and NEV patients are illustrated in Figs. 1 and 2 and summarized in Fig. 3.

The cSCC showed significantly reduced staining for claudin-1 than flat wart and normal skin epidermis, in both EV and NEV groups (p < 0.001). Claudin-1 was more often focal in *in situ* cSCC (14%) and invasive cSCC (36%) than normal skin and flat wart, which showed diffuse expression in almost 100% of the samples (Fig. 3). In order to analyze a possible simultaneous effect of the characteristics of the studied samples with the claudin-1 expression, logistic regression models were used (Table 1). In the final model, only histological type (i.e. flat wart, normal skin, *in situ* and invasive cSCC) remained significant (p < 0.001). The chance of focal claudin-1 expression is 98% smaller (1–0.02) in normal skin compared to invasive cSCC. This chance was 71.0% (1–0.29) lower in *in situ* cSCC compared to invasive cSCC.

**Table 1:** Multivariate analysis of claudins expression.

Predictors	Claudin-1 (n = 281)		Claudin-2 (n = 276)		Claudin-3 (n = 285)				Claudin-5 (n = 277)			
					Focal		Negative		Focal		Negative	
	HR^c^ (95% CI)	*P*	HR^c^ (95% CI)	*P*	HR^c^ (95% CI)	*P*	HR^c^ (95% CI)	*P*	HR^c^ (95% CI)	*P*	HR^c^ (95% CI)	*P*
EV group^a^	1.39 (0.69–2.79)	0.358	0.3 (0.08–1.07)	0.063	1.09 (0.49–2.45)	0.832	0.65 (0.24–1.76)	0.398	0.33 (0.12–0.87)	*0.025*	0.05 (0.01–0.22)	*<0.001*
Age (years)	—	—	—	—	—	—	—	—	0.98 (0.96–1.01)	0.148	0.95 (0.91–0.98)	*0.003*
Histologic type^b^		*<0.001*		*<0.001*		*<0.001*		0.678		*<0.001*		*0.014*
Flat wart	0.0 (-)	0.997	—	—	0.06 (0.02–0.20)	*<0.001*	0 (-)	0.987	3.25 (1.07–9.88)	*0.038*	41.34 (4.29–398.5)	*0.001*
Normal skin	0.02 (0.00–0.16)	*<0.001*	0(-)	0.996	0.01 (0.00–0.03)	*<0.001*	0(-)	0.986	13.06 (6.25–27.26)	*<0.001*	23.37 (2.62–208.7)	*0.005*
*In situ* cSCC	0.29 (0.12–0.71	*0,007*	0,14 (0.03–0.55)	*0.005*	0.80 (0.17–3.82)	0.781	0.36 (0.07–1.82)	0.218	1.27 (0.49–3.29)	0.619	18.64 (2.20–157,8)	*0.007*
Invasive cSCC	—	—	0.04 (0.01–0.18)	*<0.001*	—	—	—	—	—	—	—	—

^a^Reference: NEV, except for claudin-2, which EV was the reference of group.

^b^Reference: Invasive cSCC, except for claudin-2, which flat wart was the reference of histologic type.

^c^Reference: focal expression, except for claudin-3 and claudin-5, which diffuse expression was the reference.

HR: hazard rate; EV: epidermodysplasia verruciformis; NEV: non-epidermodysplasia verruciformis; cSCC: cutaneous squamous cell carcinoma.

Claudin-2 diffuse expression was seen in 93.8% of all samples studied (Fig. 3). Non-diffuse (focal and negative) expression was associated with flat wart samples (p < 0.001). After logistic regression model adjustment, histological type remained significant (p < 0.001) (Table 1). Thus, the probability of no diffuse expression was 86% lower (1–0.14) in *in situ* cSCC and 96.0% lower (1–0.04) in invasive cSCC, compared to the flat wart. Differences between EV and NEV groups have not been observed (p = 0.063).

Progressive reduction of claudin-3 expression was noticed in cSCC (p < 0.001). While diffuse expression was mostly common in normal skin samples (88.6%), focal expression was mostly common in *in situ* cSCC (63.3%) and negative expression in invasive cSCC samples (48.6%) (Fig. 3). Logistic regression model adjustment showed the histological type as significant (p < 0.001) (Table 1). Thus, the chance of focal expression is 94% lower (1–0.06) in the flat wart compared to invasive cSCC. This chance was 99.0% (1–0.01) lower in normal skin compared to invasive cSCC. In addition, the chance of focal expression in *in situ* cSCC was not distinct from invasive cSCC (p = 0.781), neither between EV and NEV groups (p = 0.832).

On the other hand, claudin-5 expression was increased in cSCC (p < 0.001). In normal skin sample, its immunostaining was frequently focal (65.4%). Flat warts were negative for this antibody in 41.0% of the cases. However, claudin-5 diffuse expression was present in most cases of *in situ* cSCC (67.3%) and invasive cSCC (84.4%). Moreover, claudin-5 diffuse expression was more common in EV (71.0%) than NEV group (49.7%) (p = 0.001) (Fig. 3). After logistic regression model adjustment, age, group (EV and NEV) and histological type remained significant in the final model (Table 1). Thus, the chance of focal expression was 67.0% (1–0.33) lower in the EV group compared to the NEV group, but it was higher in the flat wart (3.3 times) and normal skin (13.1 times greater) compared to invasive cSCC. Additionally, for each 1-year increase, a 5% (1–0.95) reduction in the chance of negative expression was observed. Besides that, the chance of negative expression was 95% lower (1–0.05) in the EV group compared to NEV group, although it demonstrated to be higher in the flat wart (41.3 times), normal skin (23.4 times) and in *in situ* cSCC (18.6 times) compared to invasive cSCC.

Diffuse expression of claudins-4, -7 and -11 was found in most specimens, in all samples studied (Fig. 3). There was no statistical difference in these claudins immunostaining between the EV and NEV groups.

## Discussion

EV is a rare inherited genodermatosis with beta-HPV susceptibility and predisposition to cutaneous carcinomas, which usually occurs during the fourth decade^2^. As described in literature, in this study, the mean age of EV patients was 44.8 years old, younger than individuals without EV (71.7 years). Although there is no difference regarding gender^1,3^, in our sample, men were more prevalent among EV patients. On the other hand, individuals without EV presented higher percentage of skin lesions on sun exposed areas, probably because chronic sun exposure is the main predisposing factor of cSCC in the general population^12^.

Beta-HPV, the infectious agent of EV, presents tropism to keratinocytes, causing cell proliferation, cellular atypia, epithelial dysplasia and cancer. In epithelial carcinogenesis, tissue architecture disappears, with intercellular disorganization and loss of cell-matrix adhesion^13^. In flat warts lesions, beta-HPV can alter the expression of E-cadherins and cytokeratin profile^14^. In carcinogenesis, changes in epidermal adhesion proteins, like tight junctions, may occur and relationships between claudins and EV skin cancer have not yet been explored. In this study, we analyzed the expression of claudins-1, -2, -3, -4, -5, -7 and -11 in flat warts and cSCC from EV patients, comparing the claudins profile in the same skin lesions from not EV patients.

We observed a strong diffuse expression of claudin-1 in normal skin and flat wart, with gradual decreasing expression in *in situ* cSCC and invasive cSCC, however not related to beta-HPV, since this downregulation occurred in EV and NEV groups. Claudin-1 down expression has already been reported regarding the progression from low-grade actinic keratoses to high-grade actinic keratoses and to cSCC^15,16^. On contrary, increase claudin-1 expression in cSCC was related in some small sample studies^17,18^. In tonsillar SCC, HPV-infected cells did not alter TJ expression^19^, as we observed in our study. Aberrant expression of claudin-1 was observed in SCC by different organs. In esophageal and breast SCC, claudin-1 down expression was associated with tumor recurrence and reduction of relapse-free survival^20,21^. Decreasing claudin-1 has been related to TJ dysfunction, with subsequent dissociation and loss of cell polarity, which may be involved in tumor progression, invasion and metastasis^16^. On the other hand, claudin-1 over expression was observed in SCC of the tongue, tonsil, nasopharynx, lung, ovary and vulva, and it was related to invasive activity of oral SCC^18,19,22–25,26^. Upregulation of claudin-1 seems to act on the invasive ability of neoplastic cells, through the activation of membrane-1-type matrix metalloproteinases (MT1-MMP) and MMP-2, resulting in greater cleavage of laminin-5-γ2153 chains. However, these mechanisms are not fully understood^26^.

Supplementary to membrane bound expression, we observed claudin-1 cytoplasmatic immunostaining in some cSCC cases. Hintsala *et al*.^15^ also described non-membranous expression of claudin-1, including nuclear location. The subcellular localization of claudin seems to be involved in its phosphorylation^27–29^. The exact role of claudin in the cytoplasm is still unknown, although some authors believe that it may be related to vesicle trafficking or to cell-matrix interactions^27^.

For claudin-2, we observed a decreased expression in flat wart lesions. Despite the fact of non-diffuse (focal or negative) expressions were more common in NEV flat wart samples, differences between the EV and NEV groups could not be established. We also have not found differences between neoplastic skin lesions and normal skin. These results are not in accordance with Hintsala *et al*.^15^, who described increased expression in cSCC and actinic keratosis, when both samples were analyzed together. As related by other authors, we also verified membranous and cytoplasmatic immunostaining of claudin-2^30,31^.

The claudin-3 immunoreactivity was described as absent in normal epidermis, as casual and weak in cSCC and as strong in Paget disease^15,30^. However, these findings are not in concordance with our results. In our series, claudin-3 expression was mostly diffuse in normal skin (80% of the cases) and its immunostaining progressively decreased from flat wart (diffuse expression in 56% of the cases), to *in situ* cSCC (focal expression in 63% of the cases) and finally to invasive cSCC, which showed no claudin-3 expression in 52% of the cases. These findings were similar in EV and NEV groups and had no association with demographic characteristics of the samples.

However, like our findings, downregulation of claudin-3 was related in other neoplastic processes. Claudin-3 down expression occurs in progression from intraepithelial vulvar to invasive neoplasia, which is a neoplastic condition often associated with HPV infection^32^. Most likely, the reduction in claudin-3 expression would be related to differentiation and metastatic progression, as described for esophageal cancer^33^.

It is interesting that in many cSCC cases, we observed nuclear immunostaining of claudin-3. Claudin-3 nuclear location has previously been reported in metastatic breast cancer and colorectal carcinoma^34,35^. There are few articles about the role of claudin in the nucleus, however its participation is speculated in cell signaling and in genetic regulation of tumor cells^34,35^. The nuclear localization of the claudin family remains to be explored in future studies.

Even though claudin-5 immunostaining has been reported as limited to endothelium^15,36^, we notice cytoplasmatic immunoreaction of this claudin in upper layers of normal epidermis, but with weak intensity. This is in line with other authors^30,37,38^, which described its positivity in *stratum granulosum*^38^. The intensity of claudin-5 expression in normal epidermis is much lower when compared to other claudins^30,38^, often requiring the use of an immunofluorescence microscope with high quality optics or confocal laser scanning microscopy^38^. This is probably the reason why claudin-5 expression is considered negative in normal skin by several authors^38^.

In cSCC, we noticed a progressive increase of claudin-5 expression from *in situ* carcinoma to invasive form. Besides this, we also observed its upregulation in EV flat warts, different from NEV group. These data suggest that beta-HPV may be involved in the initial process of cutaneous carcinogenesis, since claudin-5 increased expression has been observed in flat wart lesions from EV patients. In addition, by multivariable analysis, we found a lower chance of negative claudin-5 expression with increasing age, probably associated with the fact that skin carcinoma occurs at an older age, contrary to flat warts, which can appear in childhood.

Claudin-5 strong expression is classically described in vascular tumors, but it has also been reported in epithelial carcinomas, although with less intensity than in other claudins^30,39^. However, in cutaneous carcinomas, literature is not so concise. Claudin-5 immunoreaction was reported as weak and occasional^15^ and even with positivity just in well-differentiated and keratinized tumors areas^40^. Besides, upregulation of claudin-5 has been associated with prognostic factors, such as risk of metastases in esophageal SCC and cell proliferation and apoptosis in gastric carcinoma^31,41^.

Claudins-4, -7 and -11 have been reported to be downregulated in several human carcinomas, such as oral, colorectal and vulva. However, few studies have evaluated these claudin’s profiles in cutaneous tumors. Claudin-4 down expression was described in cSCC and its immunostaining was present only in keratinized tumor cells^15,17,42^. On the other hand, claudin-7 was overexpressed in these lesions, but not in actinic keratosis^15^. Claudin-11 expression showed decrease in mice’s skin SCC^43^. However, a study using human cell lineage, obtained from cSCC, claudin-11 was overexpressed in keratinized areas of well and moderately differentiated skin tumors, evolving with loss of its expression in undifferentiated cSCC^44^. Those results are not in accordance with our findings. For claudins-4, -7 and -11, we didn’t detect changes related to cutaneous tumorigenesis. Normal skin samples showed diffuse expression and this profile was kept in flat warts and in cSCC, in EV and NEV groups.

Despite late increasing researches about claudins and cancer, discrepant results are common and possibly associated with methodological factors. The claudins profile in epidermis may vary depending on skin thickness, since there is a possible difference in antibodies’ penetration^45^. Assessing whether a sun exposed area is also an important variable, since UVB irradiation itself can modify claudins expression in the skin^46^. Claudin immunoreaction also may vary according to the selected tumor sample, such as epidermal portion, invasive portion and tumor center, as well as keratinized areas^47,48^. Additionally, it is believed that the type of antibody used (poly or monoclonal, derived from rabbits or mice), fixation technique and tissue staining used may create artifacts, influencing immunostaining pattern^45^. Thus, standardization of the techniques used in the studies is fundamental to ensure reproducibility and precision of results.

In our series, we observed aberrant expression of claudins-1, -3 and -5 in the progression of cutaneous carcinogenesis. Possibly, these changes occur synchronously, since claudin-1 and -5 interact hetero-typically with claudin-3^9,49^. In addition, these claudins have been shown to activate pro-metalloproteinase-2, resulting in the breakdown of extracellular matrix proteins, facilitating invasion and dissemination of tumor cells^18,50^. Since we observed claudin-5 upregulation from EV flat warts to cSCC samples, it is plausible to hypothesize that beta-HPV might act at an initial stage of skin carcinogenesis, but the mechanism by which beta-HPV changes claudin expression is still unclear. Although it is known that E6 and E7 oncoproteins are both involved in beta-HPV carcinogenesis, the capacity to modify barrier epidermal adhesion proteins seems to be linked to E7. Studies using organotypic culture of skin, showed E7 gene from HPV-5 and -8 upregulated beta-catenin and ZO-1 proteins^51^, as well as E7/HPV-8 was able to alter the integrin network, promoting invasion of human keratinocytes into the underlying dermis, which was accompanied by an overexpression of extracellular matrix metalloproteinases MMP-1, MMP-8, and MT-1-MMP^52^.

Several studies support the fact that there is a relationship between aberrant claudins expression and tumors’ behavior, but the functional significance of these changes in epithelial carcinogenesis remains uncertain^53^. It is still unknown whether this aberrant expression of claudins is a cause or consequence of cancer^54^. Since this is a descriptive study, further clinical investigations are needed to determine whether claudin-5 is correlated with development and progression of cutaneous SCC in EV patients, which could allow its use as a tumor biomarker. The knowledge of how beta-HPV drives skin carcinogenesis gives opportunities for novel epithelium-targeted drug development, such as anti-claudin monoclonal antibodies, as well as new strategies for cancer therapy. Decreasing cSCC incidence would benefit not only EV patients, but also organ transplant recipients, a crescent population with a high risk of developing keratinocyte carcinoma^55^.

In conclusion, claudins-1 and -3 down expressions were observed in cSCC, but these changes could not be associated with EV. Otherwise, we found claudin-5 overexpression during progression of skin carcinoma associated with EV, which may be related to beta-HPV involvement.

## Materials and methods

### Tissue samples

We retrospectively selected 321 cutaneous tissue specimens, fixed in 10% neutral-buffered formalin and embedded in paraffin, which were previously processed by the Dermatopathology Laboratory of the University of Sao Paulo Medical School routine. These cases included 33 Bowen’s disease (*in situ* SCC), 51 invasive cSCC, 17 flat warts and 32 normal skin samples, obtained from 33 EV patients, and 30 Bowen’s disease (*in situ* SCC), 60 invasive SCC, 22 flat warts and 76 normal skin samples, obtained from 112 NEV patients. All paraffin blocks samples were sectioned and stained with hematoxylin and eosin (H&E). Normal skin tissues samples were obtained from tumors free margins resection. All the archival slides selected were evaluated for diagnosis confirmation. Clinical data were collected from patients’ records. All EV patients had clinical and evolutive characteristics of the disease. Twenty patients had been previously studied in search for evidence of infection and identification of beta-HPV type through molecular techniques^56^. Patients with other skin diseases, predisposing skin cancers (e.g. albinism, xeroderma pigmentosum and immunosuppression), as well as tumors located in palms, plants, genital and buttocks were left out of this study. Skin samples located in head/neck and limbs were considered “sun exposed area”, and skin samples from trunk were considered “not sun exposed”.

### Tissue microarray (TMA)

The cSCC samples were organized in TMA. The areas to be used in the TMA construction were marked on the H&E slide and on the donor’s block. The tumor tissues corresponding to selected areas were sampled using a manual arraying instrument (Manual Tissue Arrayer 1; Beecher Instruments, Wisconsin, USA). The sampling consisted of 2–4 malignant cores from different areas of the tumor, placed coordinately in two TMA blocks (“main” and “mirror” blocks). After the arraying was completed, TMA blocks were sectioned with a thickness of 4 μm. Four sections from different layers (one superficial, 2 medium and one from the bottom of the TMA block) were stained with H&E, to check the presence of neoplastic structures. The most representative TMA slides were submitted to claudins immunostaining.

Unlike cSCC samples, flat wart and normal skin specimens were processed by usual histological routine, since their biopsy specimens had small size, making them not suitable for TMA construction.

### Immunohistochemistry

The demonstration of claudin expression was performed by immunohistochemistry technique, according to Sadalla *et al*.^22^. Serial sections (4 μm thick) of the specimens assembled as TMA and single slides for flat wart and control skin were deparaffinized and rehydrated. Then, they were incubated in 3% aqueous hydrogen peroxide for 10 min to quench endogenous peroxidase activity. The sections were then submitted to antigen retrieval (Table 1), followed by incubation with 10% skimmed milk solution (Molico, Nestlé®) for 30 minutes at room temperature, to suppress non-specific binding of subsequent reagents. The reaction was succeeded by incubation with diluted primary antibodies (Table 2), in bovine serum albumin (BSA) fraction V (SERVA.1930) 1%, plus 0.1% sodium azide, in phosphate-buffered saline (PBS) pH 7.4, over-night at 4 °C. Then, biotinylated secondary antirabbit or antimouse antibody were used.

**Table 2:** Antibodies dilutions, antigen retrieval by moist heat and buffer systems.

Antibody	Clone	Code	Brand	Dilution	Detection system	Buffer systems	ET (min)
Claudin-1	policlonal rabbit	51–9000	Invitrogen	1:100	LSAB^a^	TRS pH 9.0	20
Claudin-2	policlonal rabbit	NBL1–09243	Novus	1:50	Novolink^b^	TRS pH 9.0	25
Claudin-3	policlonal rabbit	PA5-16867	Thermo Fisher	1:30	Novolink	TRS pH 9.0	40
Claudin-4	policlonal rabbit	Ab15104	Abcam	1:200	Novolink	TRS pH 9.0	20
Claudin-5	4C3C2 mouse	187364	Invitrogen	1:50	Picture Max^c^	TRS pH 9.0	30
Claudin-7	policlonal rabbit	3491000	Invitrogen	1:50	Novolink	TRS pH 9.0	20
Claudin-11	policlonal rabbit	SC-258711	Santa Cruz	1:50	Novolink	TRS pH 9.0	20

TRS: Target Retrieval Solution; ET: Exposure time; min: minutes.

^a^DakoCytomation, Carpinteria, CA, USA, code K0690; ^b^Leica Microsystems, Newcastle Upon Tine, United Kingdom, code K0690; ^c^Invitrogen, Carlsbad, CA, USA, code 878983).

Staining was visualized using 3,3-diaminobenzidine chromogen (Sigma Chemical Co., St. Louis, MO, USA, code D5637) 0.03% plus 1.2 ml of 3% hydrogen peroxide. The slide was counterstained with Carazzi’s hematoxylin, and mounted with Permount resin (Fisher Scientific, Fair Lawn, NJ, USA, code SP15-100) and glass coverslips. As positive controls, colon carcinoma samples were used for claudin-2 and normal skin for the other claudins. Negative controls of the reactions were obtained by replacing the primary antibody for an isotypic non-immune serum.

### Evaluation of claudin expression

Images of the TMA whole slide obtained using an Aperio AT ScanScope (Vista, CA, USA) were examined by two researchers (LLCS and MNS) using Aperio ImageScope Viewer software. The usual histological sections with normal skin and flat wart samples were analyzed with a conventional optical microscope, coupled to a digital camera to document the results.

Claudins expression was considered positive when the histological sample (normal epidermis, flat wart and cutaneous SCC) showed brown-golden coloration when the antibody was used, not considering the intensity of the reaction. The semiquantitative analysis of the results evaluated the area of staining in each core. The following scores were attributed: negative, detection in <1% of the total area of the core; focal, detection in up to 30% of the total area of the core; and diffuse, detection in >30% of the core area. In the evaluation, both membrane-bound, cytoplasmic and nuclear positivity were considered.

### Statistical analysis

The association between two categorical variables were verified using the chi-square test or Fisher’s exact test. The non-parametric Mann-Whitney test was used to compare means between two groups and the Kruskal-Wallis test for comparison of means among 3 groups. If there were differences in terms of means in the Kruskal-Wallis test, the different means in each one of those groups were identified by using the Dunn-Bonferroni multiple comparisons, in order to maintain the level of global significance. Non-parametric tests were used due to non-normality in the data distribution verified by Kolmogorov-Smirnov’s test.

To evaluate the simultaneous factors of gender, age, sun exposure area, histological type sample (normal skin, flat warts and SCC) and group (EV and NEV) (predictor variables) on each expression of claudins-1, -2, -4, -11 (dependent variant) logistic regressions were adjusted. Due to low prevalence of non-diffuse expression (focal or negative), the variables whose associations with the dependent variable were significant at 20%, in the univariate analysis, were selected for the initial models.

Then, except for the variable group (control variable), the non-significant variables at 5% were excluded one by one in order of significance (backward method). In addition, the adequacy of the final model was assessed through the Hosmer and Lemeshow test. For claudins-3 and -5, multinomial regressions have been adjusted. The claudin-7 expression was analyzed only descriptively due to the low occurrence of non-diffuse cases - 2.2% (n = 6). All statistical analyses were performed using IBM SPSS Statistics 20.0 software and *P* values lower than 0.05 were considered statistically significant.

### Ethical approval

The Institutional Review Board of the Universidade de Sao Paulo Medical School Hospital approved this study (Protocol #193.998). All procedures performed were in accordance with the Helsinki declaration of 1964 and its later amendments or comparable ethical standards. The study is based on samples obtained in the past for diagnostic purpose and retrieved from the files of the dermatopathology laboratory of the Institution. Therefore, the Institutional Review Board decided that was not necessary to acquire the informed consent from each patient^57^.
